# Supplementary material for: Identifying transposable element expression dynamics and heterogeneity during development at the single-cell level with a processing pipeline scTE
Source: Nat Commun. 2021 Mar 5;12:1456. doi: 10.1038/s41467-021-21808-x (PMC7935913; doi:10.1038/s41467-021-21808-x)
Supplement: Supplementary file 1 — Supplementary Information [file 41467_2021_21808_MOESM1_ESM.pdf]

Supplementary Fig. 1

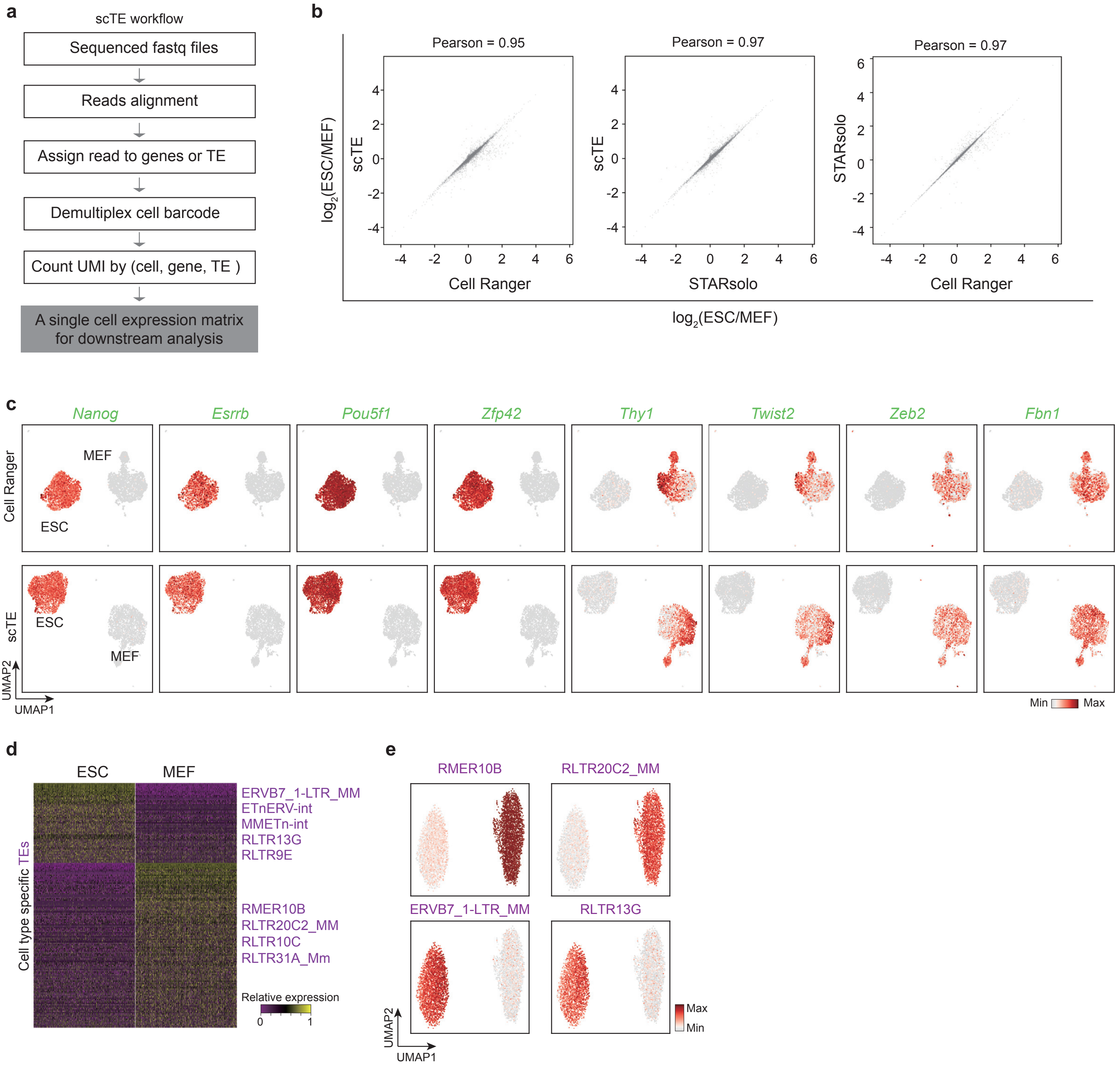

Supplementary Fig. 1 I Comparison of scTE versus Cell Ranger analysis. (**a**) Schematic describing the scTE workflow. (**b**) Scatter plot showing the gene expression correlation between scTE, Cell Ranger and STARsolo, each dot represents a single gene. (**c**) As Fig. 1b, but cells are colored by the expression of indicated genes. (**d**) Heatmap of TE expression differences between MEF and ESC single cells. Selected differentially expressed TEs are labelled. (**e**) Expression of selected TEs in a 50:50 split of MEF and ESC data.

Supplementary Fig. 2

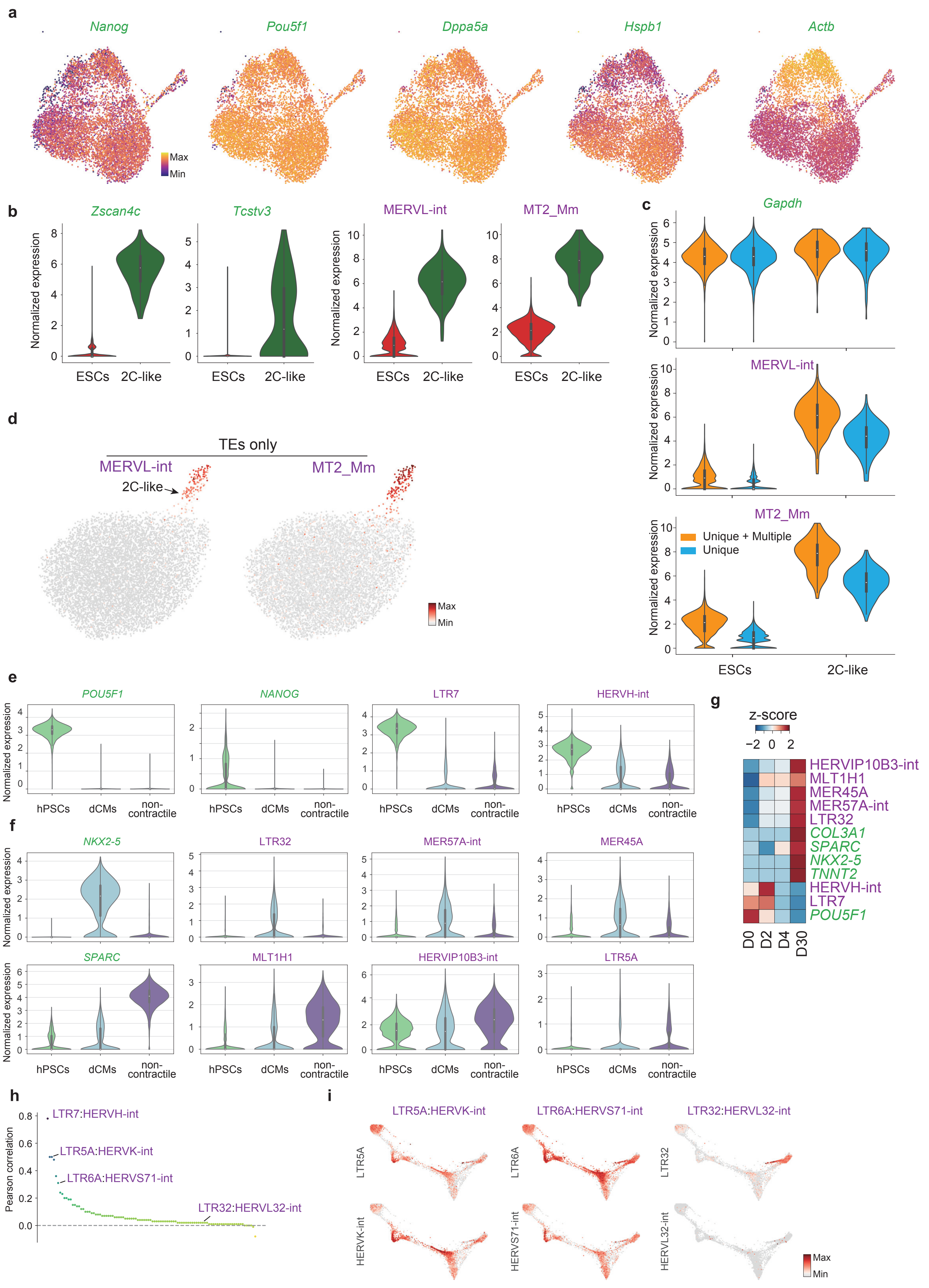

Supplementary Fig. 2 I Dynamic expression of TEs in ESCs, and in cardiac differentiation. (**a**) UMAP plots for mouse ESCs, are colored by the expression of indicated genes. (**b**) Violin plots showing the expression of 2C maker genes/TEs. Boxplots denote the medians (white dot) and the interquartile ranges (IQR). The whiskers of a boxplot are the lowest datum still within 1.5 IQR of the lower quartile and the highest datum still within 1.5 IQR of the upper quartile. n = 8133 cells examined. (**c**) Violin plots showing the expression of indicated gene/TEs with all mapped reads (unique + multiple) or only the unique mapped reads (unique). Boxplots denote the medians (white dot) and the interquartile ranges (IQR). The whiskers of a boxplot are the lowest datum still within 1.5 IQR of the lower quartile and the highest datum still within 1.5 IQR of the upper quartile. n = 8133 cells examined. n = 8133 cells examined. (**d**) UMAP plot of mouse ESCs, only TEs were used, cells are colored by MERVL expression. (**e**) Violin plots showing the expression of a selection of differentially expressed genes and TEs in hPSCs. Boxplots denote the medians (white dot) and the interquartile ranges (IQR). The whiskers of a boxplot are the lowest datum still within 1.5 IQR of the lower quartile and the highest datum still within 1.5 IQR of the upper quartile. n = 8133 cells examined. n = 17587 cells examined. (**f**) Violin plots showing the expression of a selection of genes and TEs specific to the dCM or non-contractile branch cells. Boxplots denote the medians (white dot) and the interquartile ranges (IQR). The whiskers of a boxplot are the lowest datum still within 1.5 IQR of the lower quartile and the highest datum still within 1.5 IQR of the upper quartile. n = 8133 cells examined. n = 17587 cells examined. (**g**) Expression dynamics of selected TEs and marker genes during cardiac differentiation analyzed using bulk RNA-seq. D indicates the day of the differentiation process. (**h**) Expression correlation between LTR and its internal ERV, each dot represents a single LTR and its internal ERV sequence. (**i**) As in Fig. 2d, but cells are colored by the expression level of the indicated LTR and its matching internal ERV.

Supplementary Fig. 3

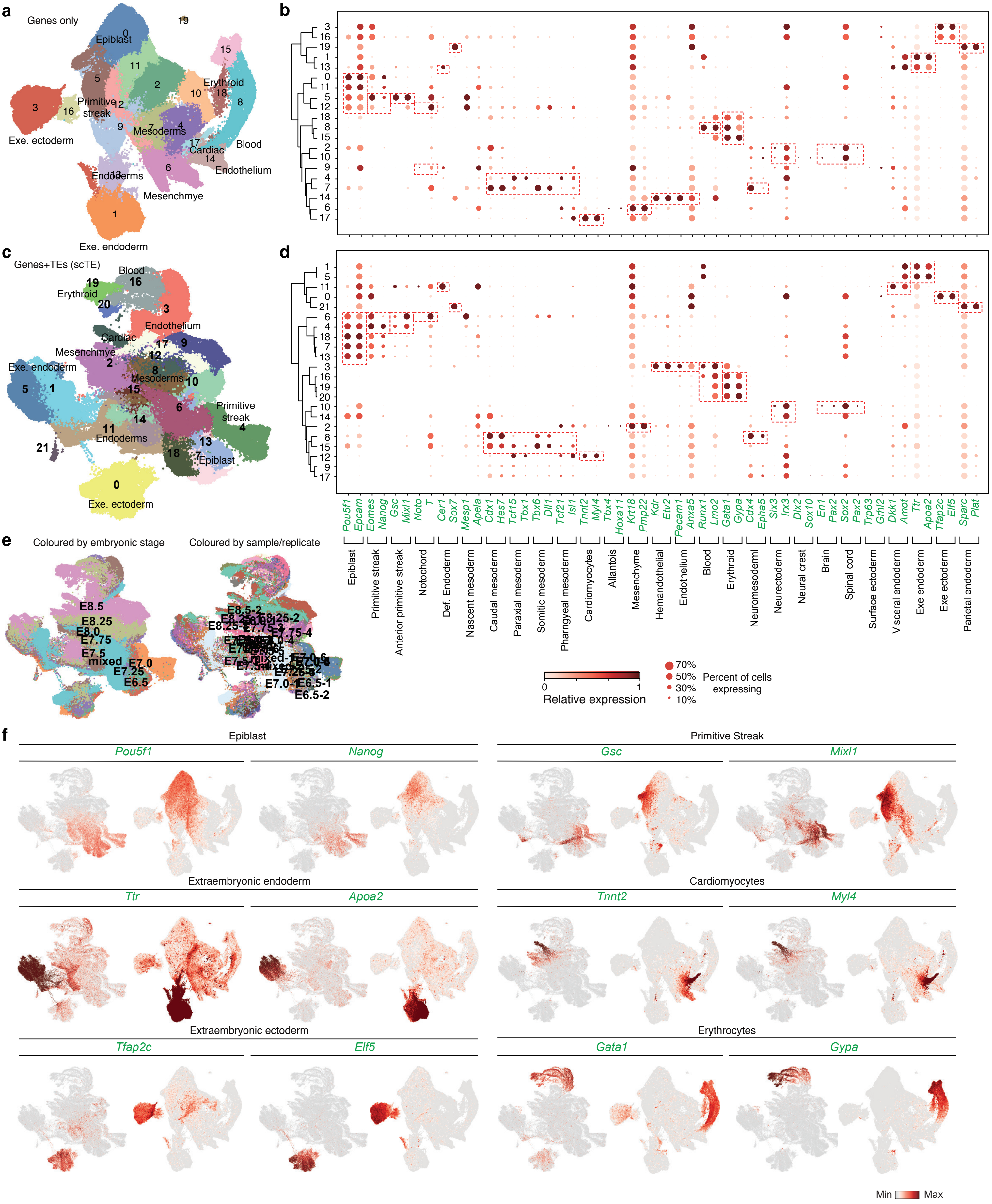

Supplementary Fig. 3 I Comparison of gene-based analysis to the gene/TE-based analysis from scTE. (**a**) Mouse gastrulation was reanalyzed using a gene-based pipeline with STARsolo. Shown are the UMAP plots, labelled with clusters using the Leiden algorithm (resolution=0.5). Selected lineages are indicated. (**b**) Dot plots showing the expression level and the indicated percent of cells the gene is expressed in, for the indicated marker genes. This panel shares the x-axis with panel d. (**c**) Analysis of the gastrulation data using scTE. Cells were plotted by UMAP, with Leiden grouping (resolution=0.5). Selected lineages are labelled. (**d**) Dot plot showing the expression of selected marker genes, as in panel b. Panels b and d share axes and legends. (**e**) UMAP plot of the scTE-analyzed gastrulation data, colored by the embryonic stage, or by the sample/replicate. (**f**) A selection of UMAP plots showing the expression of the indicated marker genes in the gene+TE-based analysis (left), or the gene-based analysis (right).

Supplementary Fig. 4

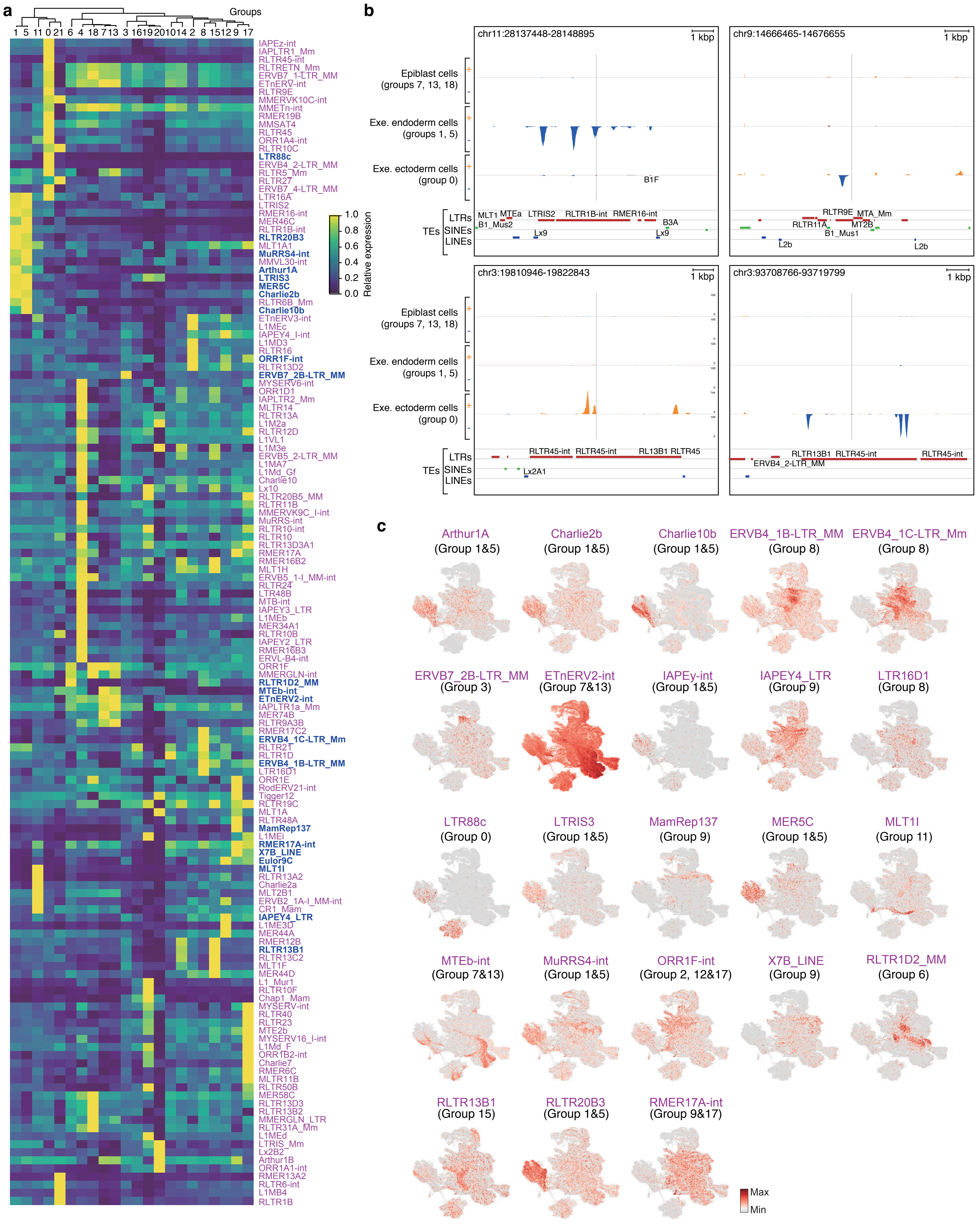

Supplementary Fig. 4 l Expression of TEs in the gastrulation data. (**a**) Heatmap showing the expression level of the indicated TEs in the groups, as defined in Supplementary Fig. 3c. Expression was transformed into the unit variance, and the groups were clustered. The TEs shown here were significantly different (Benjamini-Hochberg corrected Wilcoxon rank-sum test, p-value<0.01), and at least >2-fold change between groups. (**b**) Genome views showing example individual loci. The reads from the gastrulation scRNA-seq data were split according to the groups corresponding to the epiblast, extraembryonic endoderm, or extra embryonic ectoderm cells, as defined in Supplementary Fig. 3c. Shown here are selected genome views indicating the 3' expression of extraembryonic endoderm, or ectoderm-specific TEs.'+' and '-' indicates the strand. Care should be taken in the interpretation of these genome views, and we show them for illustrative purposes only, to show typical read distribution from the 10x data. (**c**) UMAP plots for a selection of TEs, as labelled in blue in panel a.

Supplementary Fig. 5

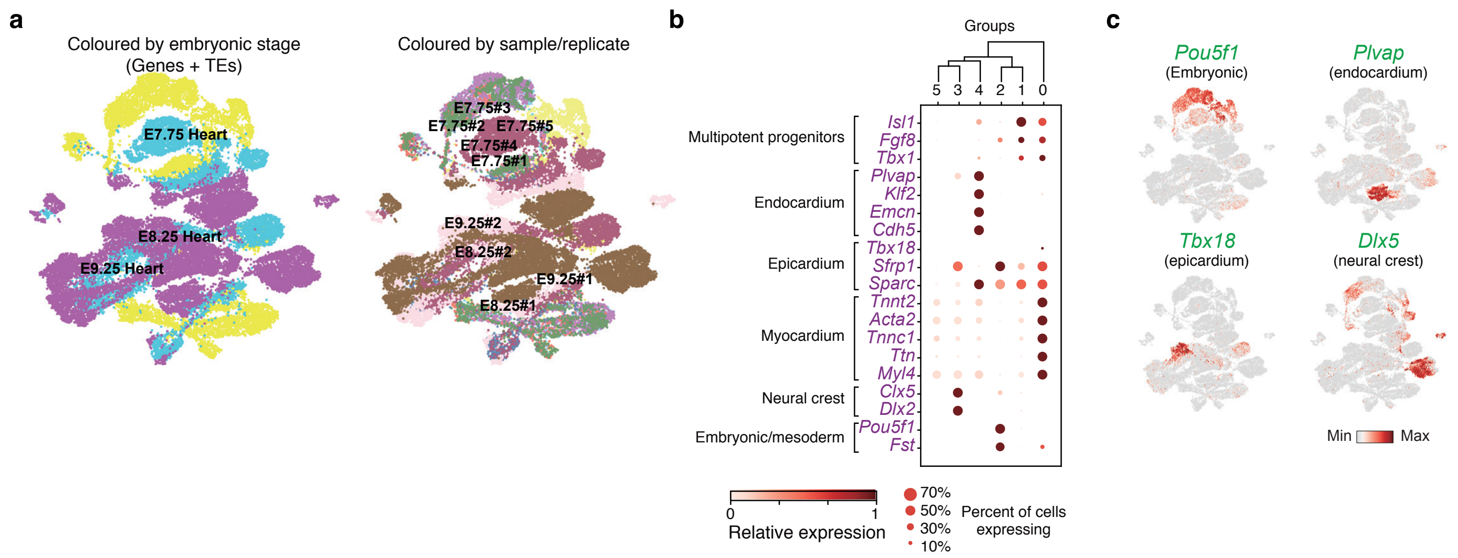

Supplementary Fig. 5 | Detection of TEs in the embryonic heart scRNA-seq dataset. (**a**) UMAP plot of embryonic heart scRNA-seq data, colored by embryonic stage (left) or by sample/replicate (right). (**b**) Expression of the indicated marker genes in the clusters as defined in Fig. 3i. Color indicates the normalized expression. The size of the dot indicates the percent of cells expressing that gene. (**c**) UMAP plots of selected marker genes, from the indicated lineages.

Supplementary Fig. 6

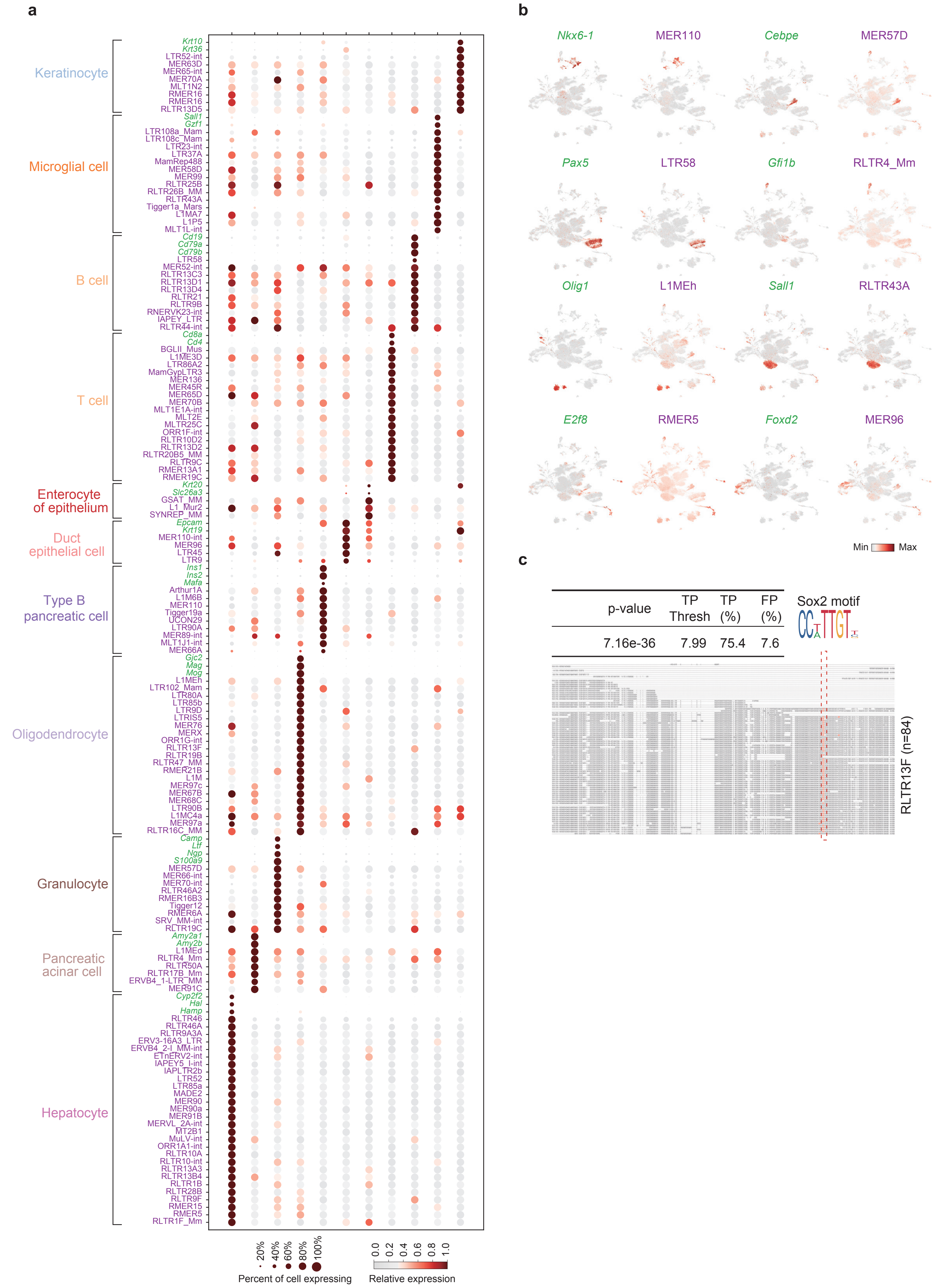

p-value	TP Thresh	TP (%)	FP (%)
7.16e-36	7.99	75.4	7.6

Supplementary Fig. 6 I Cell-type specific expression of TEs. (**a**) Dotplot showing the expression (color) and percentage of cells expressing the indicated marker genes for the indicated groups. (**b**) UMAP plots showing the expression of selected cell type-specific TFs andTEs. (**c**) Upper panel: Significantly enriched transcription factor binding motifs in the RLTR13F TEs. The motif analyses was measured using AME with one-tailed Fisher's Exact test from the MEME suite; Lower panel: Alignment of all of the genomic copies of the RLTR13F, showing the location of the SOX2 motif in red. The consensus SOX2 sequence logo is indicated at the top of the TE. The number of copies of the TE are indicated (n=84).

Supplementary Fig. 7

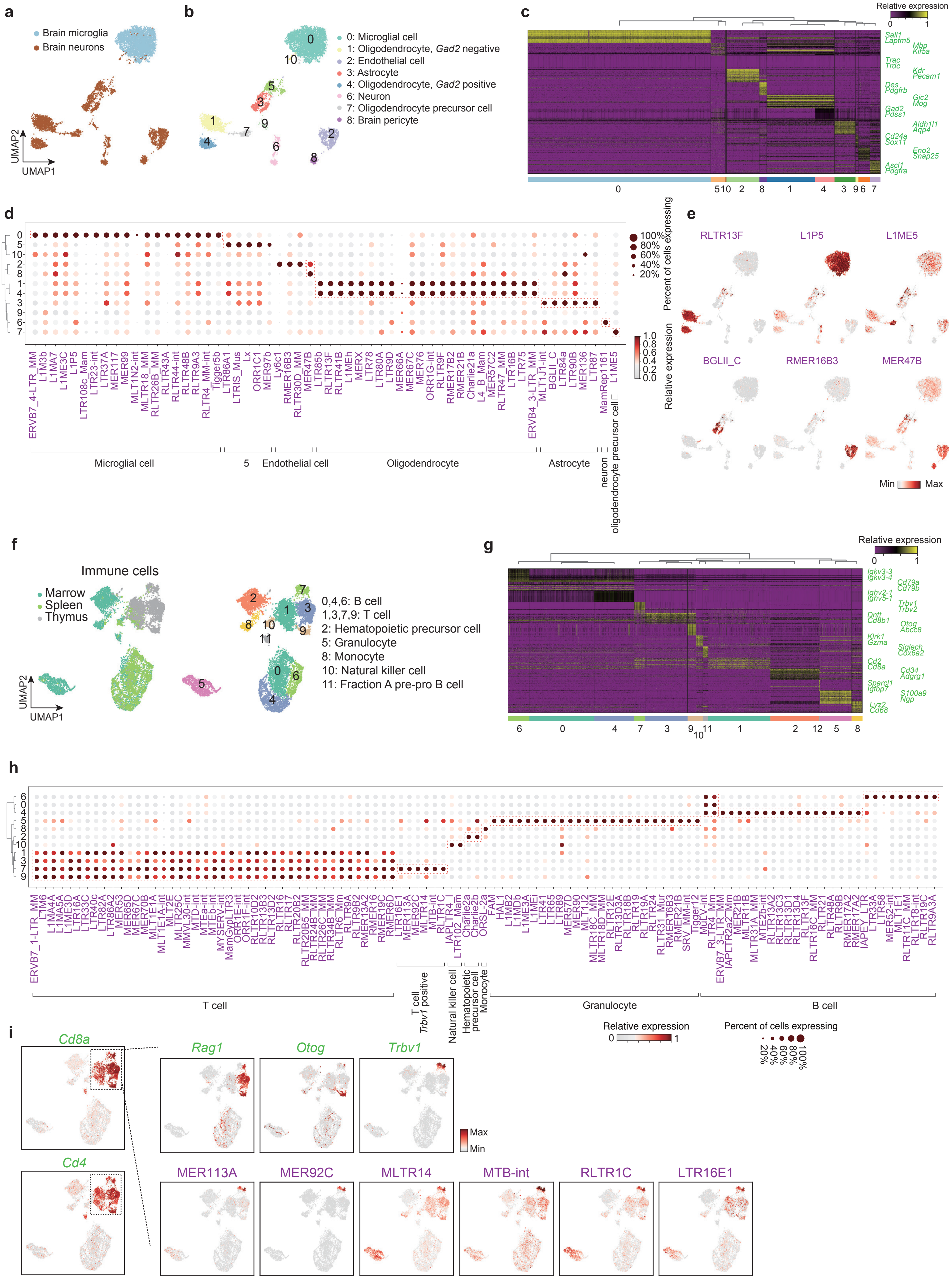

Supplementary Fig. 7 I TEs are widely expressed in mouse brain and immune system. (**a**) UMAP plot of mouse brain microglia and neurons. (**b**) UMAP plot as in panel a, but clustered into groups (Leiden, resolution=0.5). The indicated cell types are labelled according to the known marker genes from panel d. (**c**) A gene expression heatmap showing the top differentially expressed genes for each cell cluster from panel b. (**d**) Dotplot showing the expression (color) and percentage of cells expressing the indicated TEs, in the indicated groups from panel b. (**e**) UMAP plots showing the indicated TE expression across cell types. (**f**) UMAP plot of mouse marrow, spleen and thymus tissues. (**g**) A gene expression heatmap showing the top differentially expressed genes for each cell cluster as defined in panel f. Selected marker genes are indicated on the right-hand side. (**h**) Dot plot showing the expression (color) and percentage of cells expressing the indicated TEs, in the indicated groups from panel f. (**i**) UMAP plot showing the indicated gene and TE expression for T cell-specific genes/TEs

Supplementary Fig. 8

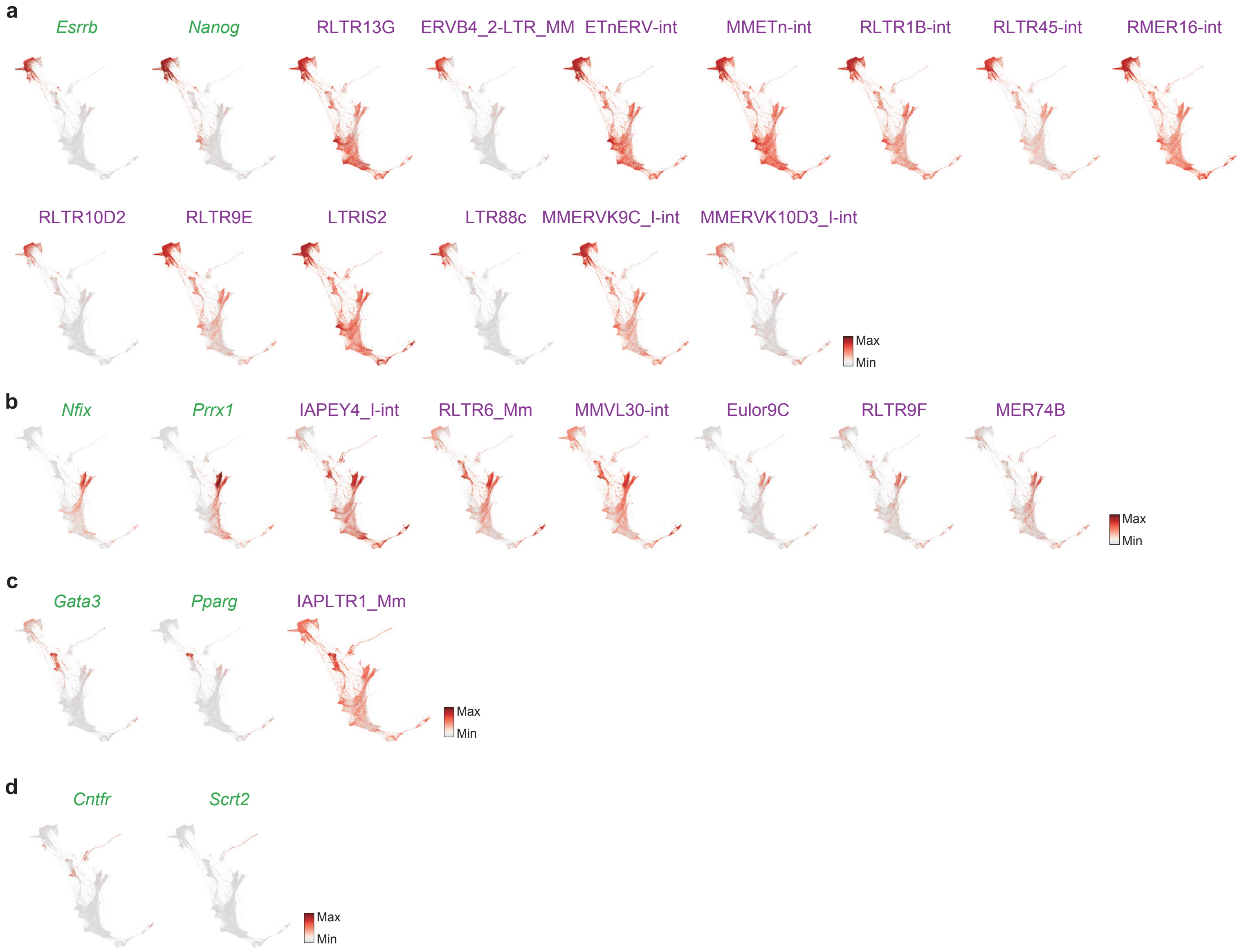

Supplementary Fig. 8 | Dynamic expression of TEs during OKSM induced somatic cell reprogramming. (**a**) Visualization of scRNA-seq profiles for OKSM-based reprogramming, cells are colored by the expression of pluripotency marker genes and selected TEs. (**b**) Expression of stromal marker genes and selected TEs. (**c**) Expression of trophoblast marker genes and the TE IAPLTR1_Mm. (**d**) Expression of neural marker genes.

Supplementary Fig. 9

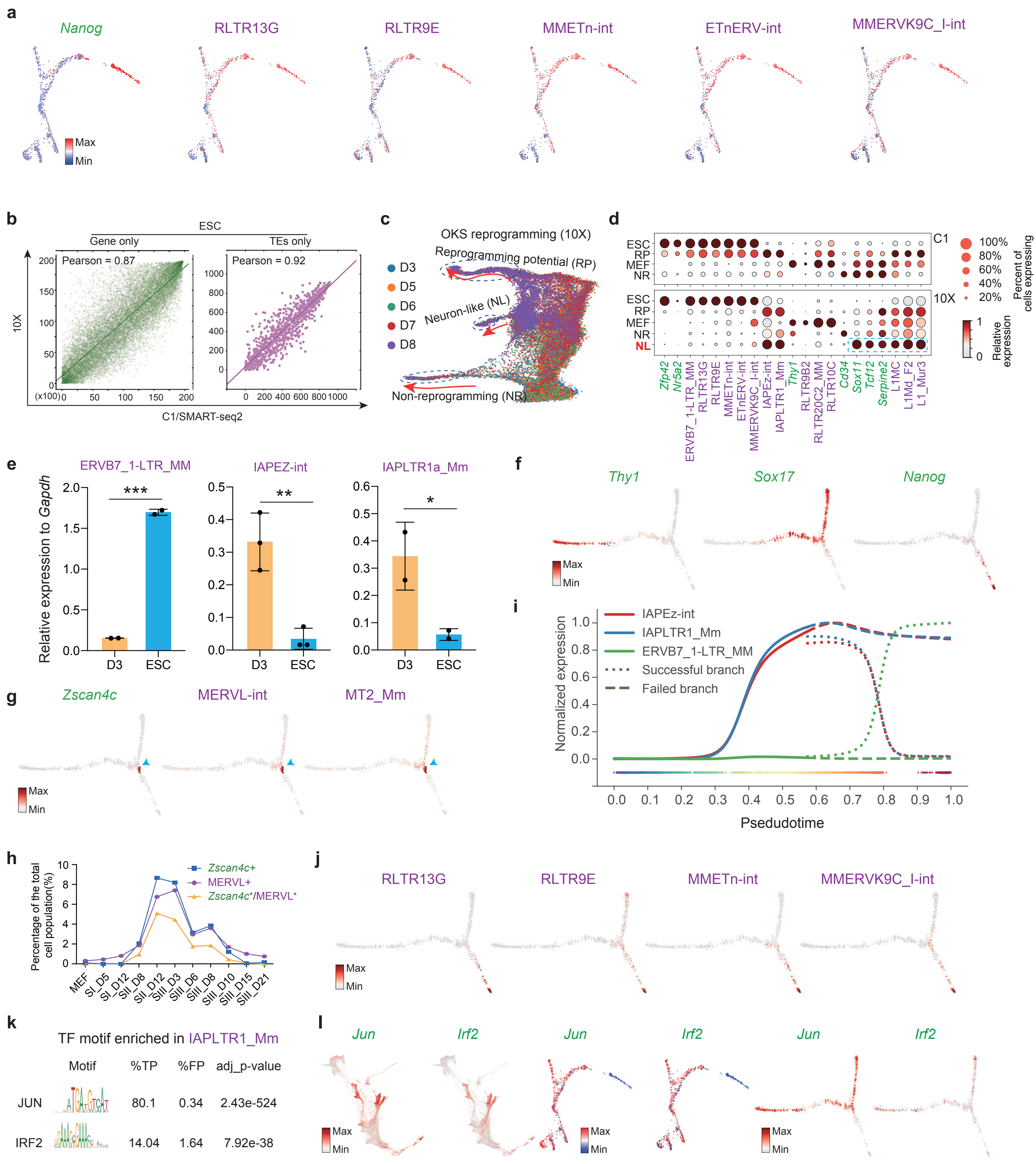

Motif		%TP	%FP	adj_p-value
JUN		80.1	0.34	2.43e-524
IRF2		14.04	1.64	7.92e-38

Supplementary Fig. 9 l Dynamic expression of TEs during OKS and chemical induced somatic cell reprogramming. (**a**) Expression of the pluripotent marker gene *Nanog* or TEs during OKS reprogramming. **(b)** Expression correlation analysis of genes (left) and TEs (right) between 10x and C1 platform for mouse ESCs. Genes or TEs were ranked by expression measure. Each dot represents a single gene or TE. (**c**) UMAP layout of cells during OKS reprogramming from 10x protocol, cells are colored by time point. (**d**) Dotplot showing the expression of selected genes and TEs in the indicated branch of cell types. (**e**) Scatter plot show the qRT-PCR analysis the expression of selected TEs. Primers used are described in **Supplementary Table 2**. Data are presented as mean values +/- SD; (**f**) Expression of marker genes during chemical reprogramming. (**g**) As in panel g, but showing the expression of 2C-like related genes or TEs. (**h**) Percent of 2C-like cells at different time points during chemical reprogramming. (**i**) Expression kinetics of selected maker TEs along pseudotime during reprogramming. (**j**) Expression dynamics of the indicated TEs during chemical reprogramming. (**k**) Significantly enriched transcription factor binding motifs in the IAPLTR1_Mm TEs. The motif analyses were measured using AME one-tailed Fisher's Exact test from the MEME suite. (**l**) Expression level of *Jun* and *Irf2* in the three different reprogramming systems, OKSM (left), OKS (middle) and chemical-reprogramming (right).

Supplementary Fig. 10

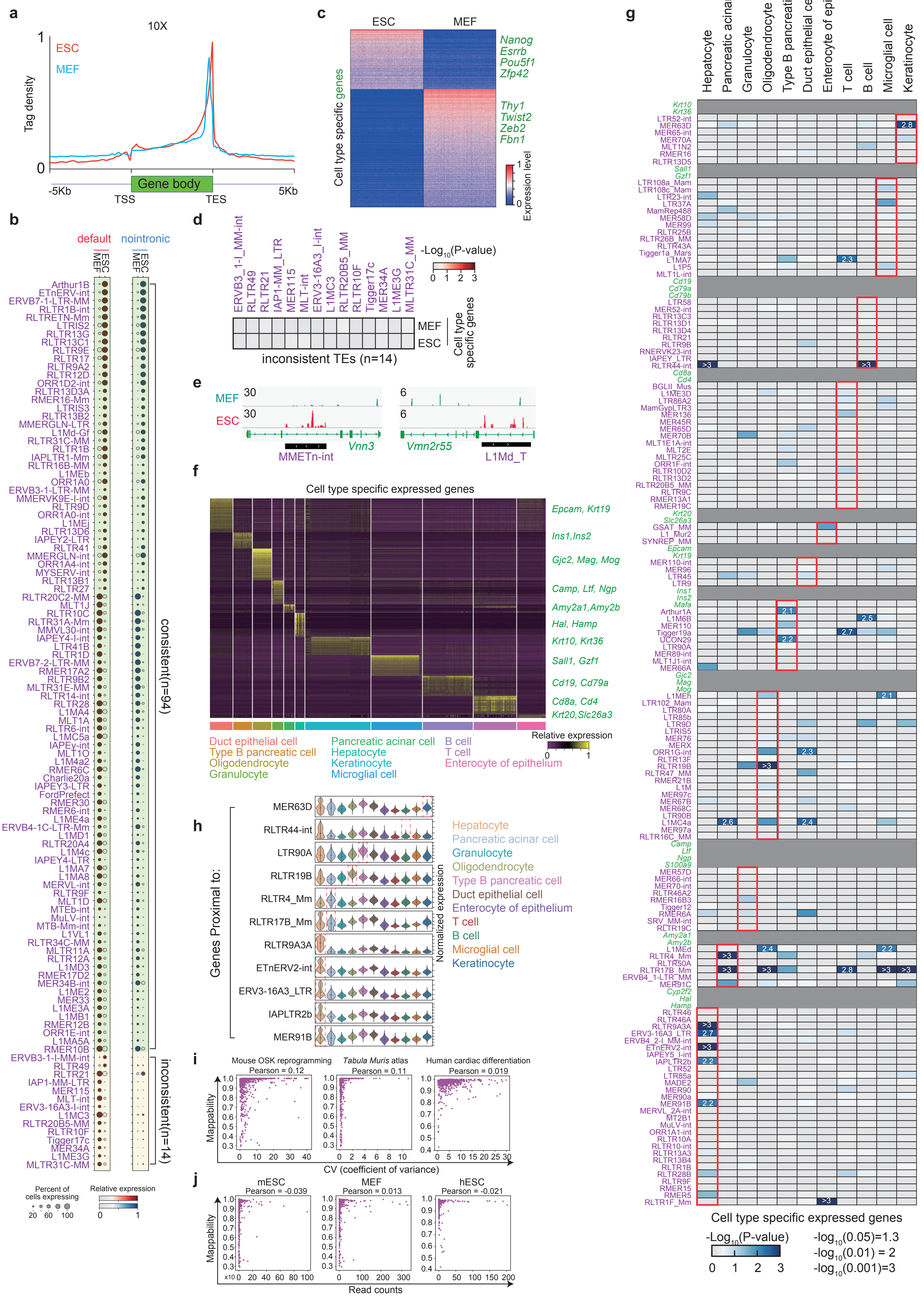

Supplementary Fig. 10 | Intronic reads rarely influence the cell type-specific designation of TEs in whole-cell scRNA-seq data. (a) Read density across gene bodies, and the flanking 5 kb regions. (**b**) Dotplot showing the normalized expression of TEs, using either the default 'exclusive' mode (count reads first to exons, and then secondly to TEs), or the mode 'nointronic' (discards all TE reads that are inside an intron of a gene, even if that gene is not expressed). Note that the mapping of reads to the genome was identical in both modes. (**c**) Heatmap of cell type-specific expressed genes for MEFs and ESCs. Each column is a cell, each row is a gene. (**d**) Cell type-specific genes (panel c) and the genomic location of all copies of the cell type-specific 'inconsistent' TEs (panel b) were collected. The relationship between cell type-specific TEs (column) and cell type-specific gene sets (row) was determined by measuring the significance of the overlap of TEs versus the set of cell type-specific genes using bedtools fisher, if the genes/TEs are physically proximal and co-regulated, then the P-value should be significant. P-values were calculated using one-tailed Fisher's Exact test by bedtools. (**e**) Example of intronic TE expressed inside the non-expressed genes. (**f**) Cell type specific expressed genes in *Tabula Muris* somatic cells. (**g**) Similar to panel d, correlation analysis for cell type specific genes (columns) and TEs (rows). The row order is the same as Supplementary Fig. 6a. Cell type-specific genes are shown as examples and are marked in green. P-values were calculated using one-tailed Fisher's Exact test by bedtools. (**h**) Violin plot showing the expression of genes proximal to (within 2kb) the indicated TEs across different cell types. (**i**) Mappability was generated for each TE family by defining mappability as the mean of all 1/N for each 90 bp window (and flanking 60 bp) in each genomic copy of the TE, where N is the number of times that 90bp window was found in the mouse or human genome. This mappability score simulates the mappability for 10x-style data. Scatter plots indicate the mappability versus the coefficient of variance (CV), and each dot is a TE type. Pearson correlations are indicated. (**j**) As in panel i, but mappability versus read count for each TE type.

Supplementary Fig. 11

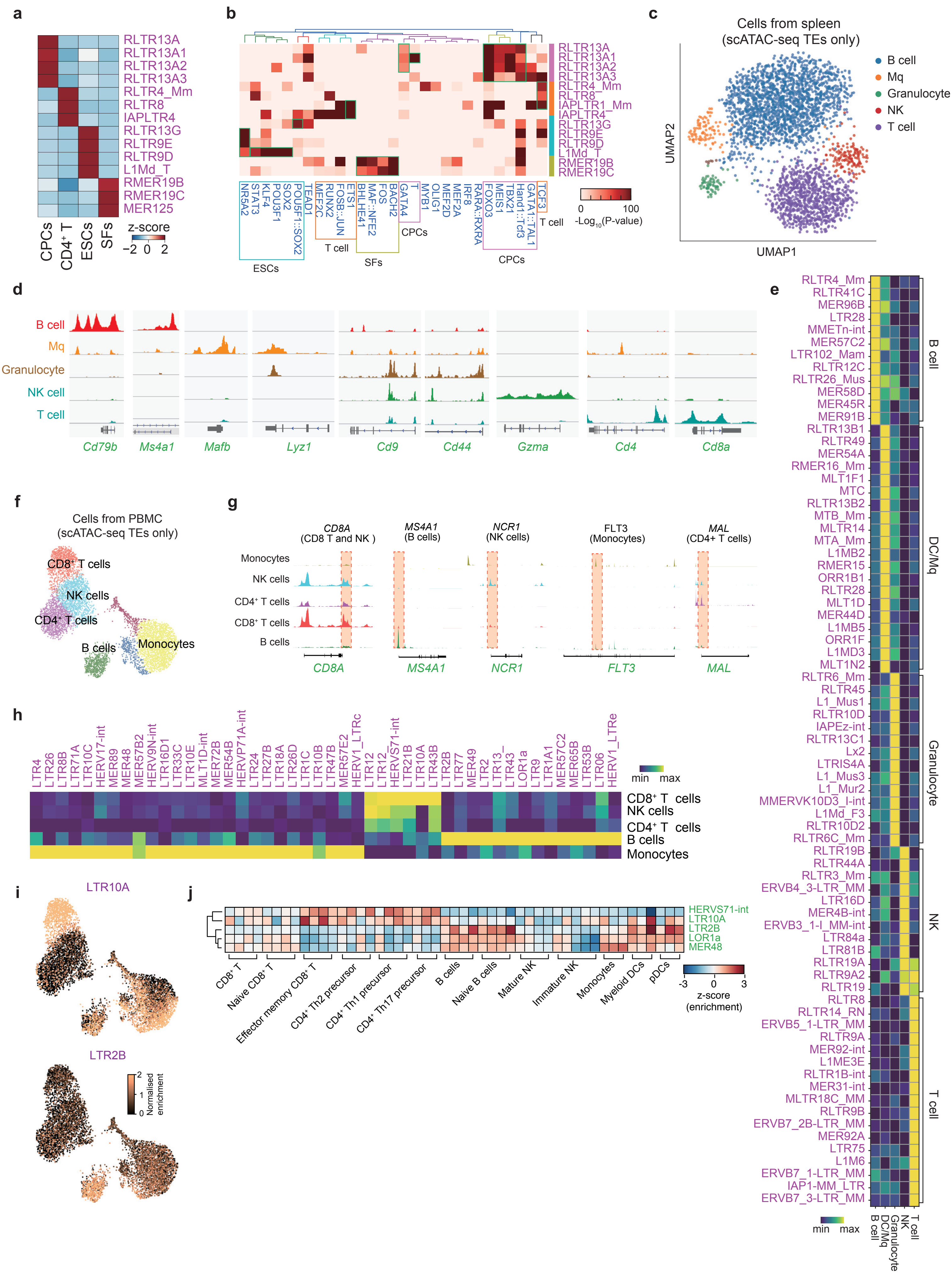

Supplementary Fig. 11 | Analysis of the Single-Cell ATAC-seq data. (**a**) Heatmap showing the enrichment of selected TEs across cell types in pseudo-bulk ATAC-seq data generated from the scRNA-seq. (**b**) Heatmap showing TF motif enrichment in selected TEs. The motif analyses was measured using AME with one-tailed Fisher's Exact test from the MEME suite. (**c**) UMAP plot of the TE chromatin state from mouse spleen scATAC-seq data, cells were colored by cell types. (**d**) Genome track plots showing the aggregated scATAC-seq profiles of selected marker genes for the indicated lineages from panel c. (**e**) Heatmap of significantly differentially open TEs between the indicated cell types from panel c. The TEs shown here were significantly different (Benjamini-Hochberg corrected Wilcoxon rank-sum test, p-value<0.01), and at least >2-fold change between groups. (**f**) UMAP plot of the TE chromatin state from PBMC scATAC-seq data, cells were colored by cell types. (Leiden clustering, resolution=0.8). Cell types were annotated based on the specific opened marker genes (See panel d). Data was from 10x genomics website. (**g**) Genome track plots showing the aggregated scATAC-seq profiles of selected marker genes for the indicated lineages. (**h**) Heatmap of significantly differentially open TEs between the indicated cell types. (Benjamini-Hochberg corrected Wilcoxon rank-sum test, p-value<0.01), and at least >2 fold change between groups. (**i**) UMAP plot, as panel c, but cells are colored by expression of the indicated TEs. (**j**) Heatmap showing a z-score enrichment of selected TEs across different immune cell types from bulk ATAC-seq data.

Supplementary Fig. 12

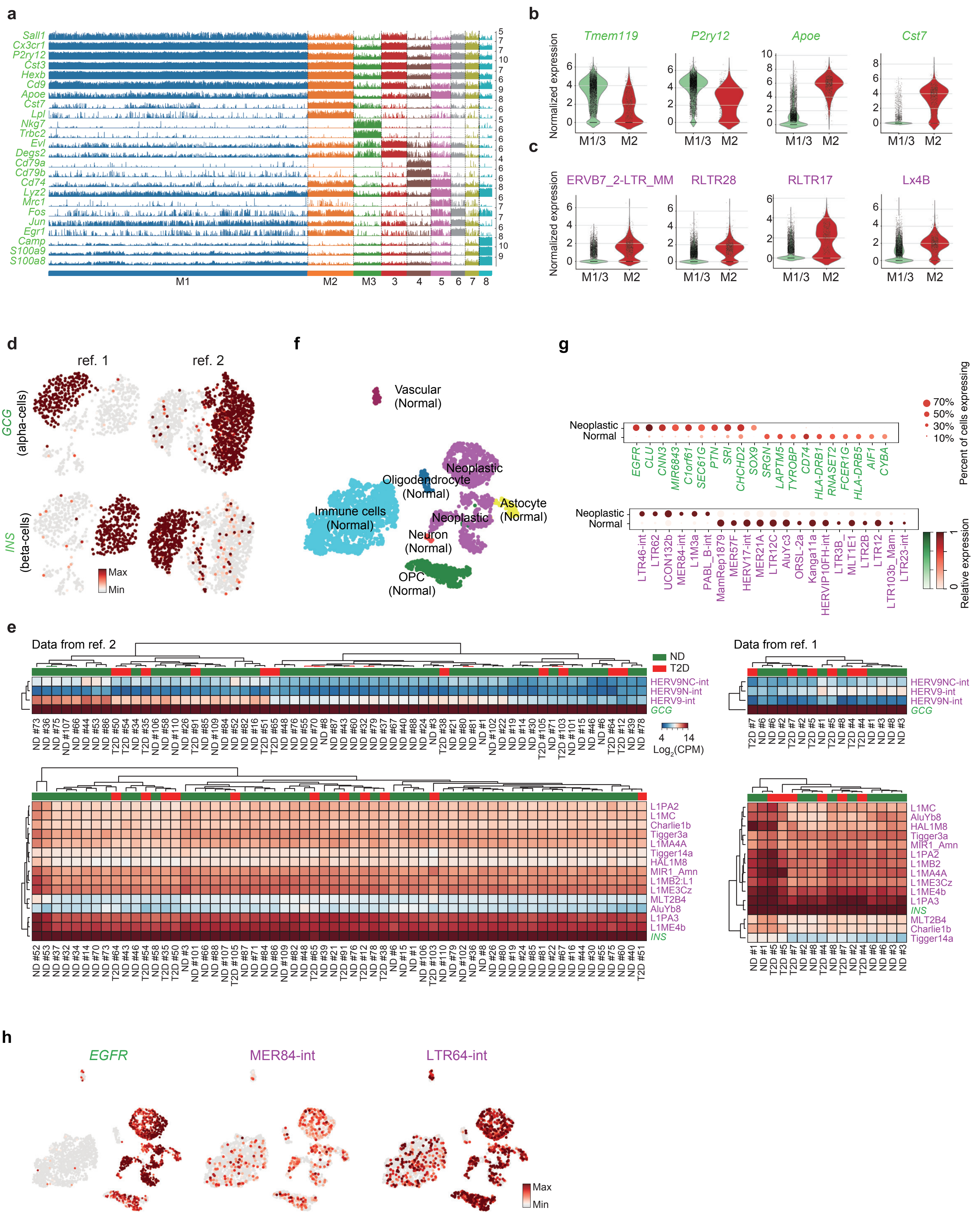

Supplementary Fig. 12 | TE and marker gene expression in a mouse model of Alzheimer's disease, in human type 2 diabetes and glioblastoma. (**a**) Tracksplot showing the known marker gene expression for cell clusters, the cell clusters are from **Fig. 7b**. The x-axis is for cells, each column represents an individual cell, and the y-axis represent the expression value. (**b**) Violin plots showing the expression of known marker genes. (**c**) Violin plots, as in panel b, showing the expression of indicated TEs. (**d**) UMAP plots showing the indicated marker gene expression for alpha and beta cells. The yellow represents high expressed and black represent low expressed. (**e**) Heatmap of expression for the indicated genes and TEs in bulk RNA-seq data. The '#' number indicates the patient, and T2D=type 2 diabetes and ND=Non-diabetic. (**f**) UMAP plot of human glioblastoma, the metadata provided in the original study was used to label the cell identity. (**g**) Dot plot showing the differentially expressed genes and TEs between normal and neoplastic group cells. (**h**) UMAP plots showing the expression of selected TEs and *EGFR*, a known glioblastoma neoplastic gene.

Supplementary Table 1. Summary of datasets utilized in this manuscript.

Type	Condition	Accession
scRNA-seq	Mouse E6.5-E8.5	https://www.ncbi.nlm.nih.gov/geo/query/acc.cgi?acc=GSE87038
	Mouse E7.75-E9.25	https://www.ncbi.nlm.nih.gov/geo/query/acc.cgi?acc=GSE126128
	Tabula Muris 20 organs	https://www.ncbi.nlm.nih.gov/geo/query/acc.cgi?acc=GSE109774
	OKS reprogramming	https://www.ncbi.nlm.nih.gov/geo/query/acc.cgi?acc=GSE103221
	Chemical reprogramming	https://www.ncbi.nlm.nih.gov/geo/query/acc.cgi?acc=GSE114952
	OKSM reprogramming	https://www.ncbi.nlm.nih.gov/geo/query/acc.cgi?acc=GSE122662
	Cardiomyocytes differentiation	https://www.ebi.ac.uk/arrayexpress/experiments/E-MTAB-6268/
	Alzheimer's Disease	https://www.ncbi.nlm.nih.gov/geo/query/acc.cgi?acc=GSE98971
	Human type 2 diabetes	https://www.ncbi.nlm.nih.gov/geo/query/acc.cgi?acc=GSE81608
	Human type 2 diabetes	https://www.ncbi.nlm.nih.gov/geo/query/acc.cgi?acc=GSE86473
	Human glioblastoma	https://www.ncbi.nlm.nih.gov/geo/query/acc.cgi?acc=GSE84465
Bulk RNA-seq	Mouse TSC	https://www.ncbi.nlm.nih.gov/geo/query/acc.cgi?acc=GSE122217
	Mouse XEN	https://www.ncbi.nlm.nih.gov/geo/query/acc.cgi?acc=GSE106158
	Mouse ESC	https://www.ncbi.nlm.nih.gov/geo/query/acc.cgi?acc=GSE57409
	Cardiomyocytes differentiation	https://www.ncbi.nlm.nih.gov/geo/query/acc.cgi?acc=GSE85332
	Human type 2 diabetes	https://www.ncbi.nlm.nih.gov/geo/query/acc.cgi?acc=GSE86473
	Human type 2 diabetes	https://www.ncbi.nlm.nih.gov/geo/query/acc.cgi?acc=GSE50398
scATAC-seq	Mouse cell line and spleen	https://www.ebi.ac.uk/arrayexpress/experiments/E-MTAB-6714/
	Human PBMC	https://support.10xgenomics.com/single-cell-atac/datasets/1.1.0/atac_pbmc_10k_v1
Bulk ATAC-seq	Human immune cells	https://www.ncbi.nlm.nih.gov/geo/query/acc.cgi?acc=GSE118189
ChIP-seq	TCF7	https://www.ncbi.nlm.nih.gov/geo/query/acc.cgi?acc=GSE31221
	SOX2	https://www.ncbi.nlm.nih.gov/geo/query/acc.cgi?acc=GSE113913
	TFAP2C	https://www.ncbi.nlm.nih.gov/geo/query/acc.cgi?acc=GSE51511

Supplementary Table 2. qRT-PCR primer sequences (Related to Supplementary Fig. 9e)

TEs name	Primer (5'-3')
ERVB7_1-LTR_MM#1-F	TTCCTGCTCATTCGTTCTG
ERVB7_1-LTR_MM#1-R	CCGCCGAATAATCTCTGG
ERVB7_1-LTR_MM#2-F	TCCTGCTCATTCGTTCTG
ERVB7_1-LTR_MM#2-R	TCCCGCCGAATAATCTCT
IAPEZ-int#1-F	GGGAAATGATTTGGCAGATAAGG
IAPEZ-int#1-R	ATGAGGAACTGGCAAGAACT
IAPEZ-int#2-F	CGCCCGTGACATTGTTACT
IAPEZ-int#2-R	CCATTTGCCAGACCTGTAGAG
IAPEZ-int#3-F	GCCCAAACTAGGAGAGACAAG
IAPEZ-int#3-R	CCAGCGACCTATTGCCTAAG
IAPLTR1a_Mm#1-F	GCTGTGTTCTAAGTGGTA
IAPLTR1a_Mm#1-R	AGAATTATCCTTCGCCTAG
IAPLTR1a_Mm#2-F	GCAGCCAATCAGGGAGTG
IAPLTR1a_Mm#2-R	AAGAACGCAACAGACCAGAATC

Supplementary references

1.
Lawlor, N. *et al.* Single-cell transcriptomes identify human islet cell signatures and reveal cell-type-specific expression changes in type 2 diabetes. *Genome Res* **27**, 208-222, doi:10.1101/gr.212720.116 (2017).

2.
Fadista, J. *et al.* Global genomic and transcriptomic analysis of human pancreatic islets reveals novel genes influencing glucose metabolism. *Proc Natl Acad Sci U S A* **111**, 13924-13929, doi:10.1073/pnas.1402665111 (2014).
